# Controllable Preparation of Si_3_N_4_@MgSiN_2_ Core–Shell Powders via a “Template Growth” Mechanism in NaCl-KCl Mixed Molten Salt

**DOI:** 10.3390/ma19071475

**Published:** 2026-04-07

**Authors:** Yiming Liu, Weiming Wang, Yong Mo, Lei Guo, Zheng Peng, Weide Wang, Qingsong Ma

**Affiliations:** Science and Technology on Advanced Ceramic Fibers and Composites Laboratory, College of Aerospace Science and Engineering, National University of Defense Technology, Changsha 410073, China

**Keywords:** Si_3_N_4_, core–shell powders, molten salt method, in-situ reaction, MgSiN_2_

## Abstract

Si_3_N_4_@MgSiN_2_ composite powder with a core–shell structure was successfully synthesized via the in situ reaction between Mg and α-Si_3_N_4_ using a NaCl–KCl mixed molten salt in this study. The effects of process parameters, including the molten salt system, reaction temperature, and Mg/Si_3_N_4_ mass ratio, on the morphology, phase composition, and microstructure of the coating layer were investigated. The results indicate that the reaction follows a “template growth” mechanism. Mg-containing species dissolve in the molten salt, diffuse to the surface of Si_3_N_4_ particles, and react with α-Si_3_N_4_, resulting in a relatively uniform MgSiN_2_ layer at 1300 °C. The yield of MgSiN_2_ layer exhibits a linear positive correlation with the Mg/Si_3_N_4_ mass ratio, enabling controllable microstructural regulation through adjustment of the starting materials composition. The core–shell powder forms a liquid phase at a relatively low temperature (approximately 1350 °C), demonstrating excellent sintering activity. This work provides a new material foundation for the fabrication of silicon nitride ceramics with high thermal conductivity.

## 1. Introduction

Silicon nitride (Si_3_N_4_) ceramics have been widely used in fields such as aerospace, chemical engineering, and metallurgy owing to their excellent chemical stability and mechanical properties [1,2,3]. Meanwhile, β-Si_3_N_4_ exhibits great potential as a new generation of high-performance electronic packaging and thermal management material, due to its theoretically high thermal conductivity exceeding 320 W·m^−1^·K^−1^ [4]. However, translating this potential into practical performance faces significant challenges in material processing. Si_3_N_4_, as a strong covalent compound, possesses an extremely low atomic self-diffusion coefficient, making full densification via conventional solid-state sintering difficult [5]. The common approach is liquid-phase sintering using sintering aids such as Y_2_O_3_ and MgO [6,7]. At elevated temperatures, these aids react with the SiO_2_ present on the surface of α-Si_3_N_4_ particles to form an oxynitride liquid phase [8]. This liquid phase provides a fast pathway for mass transport, thereby driving the densification process and promoting the α → β phase transformation [9]. Nevertheless, during the liquid-phase sintering process, oxygen atoms dissolve into the β-Si_3_N_4_ lattice to form silicon vacancies, triggering strong phonon scattering and significantly reducing the phonon mean free path, which is a key factor limiting the thermal conductivity of Si_3_N_4_ ceramic [10,11]. In addition, the liquid phase will remain in the form of low thermal conductivity amorphous glass phase at grain boundaries and polycrystalline intersections after cooling, becoming a barrier for phonon transmission [12].

During the sintering of Si_3_N_4_ ceramics, the dissolution of α-Si_3_N_4_, the nucleation of β- Si_3_N_4_, and the growth of β-Si_3_N_4_ grains all occur within the liquid phase [13]. Reducing the oxygen content in the liquid phase can directly decrease oxygen impurities within the β-Si_3_N_4_ lattice, thereby enhancing thermal conductivity. Consequently, constructing a low-oxygen liquid phase has become an effective approach for improving thermal conductivity. In the context where significant breakthroughs in low-oxygen α-Si_3_N_4_ powder have not yet been achieved, the use of non-oxide sintering aids to replace traditional oxide aids has become a mainstream research direction. Currently, non-oxide aids under investigation include: silicides (ZrSi_2_ [14], MgSi_2_ [15,16]), fluorides (YbF_3_ [17,18], YF_3_ [19], MgF_2_ [19,20,21], LiF [22,23]), hydrides (YH_2_ [24], GdH_2_ [25], ZrH_2_ [26]), and nitrides (Y_2_Si_4_N_6_C [27], YN [28], Mg_3_N_2_ [15], MgSiN_2_ [29,30,31,32,33]). Non-oxide aids can establish a high-nitrogen, low-oxygen liquid phase, significantly reducing the oxygen content dissolved in β-Si_3_N_4_ while promoting phase transformation and the growth of β-Si_3_N_4_ grains, all of which are beneficial for improving the thermal conductivity of Si_3_N_4_ ceramics. Among the various types of non-oxide aids, nitrides exhibit excellent compatibility with Si_3_N_4_. Notably, magnesium silicon nitride (MgSiN_2_), as a novel oxygen-free nitride, has demonstrated exceptional effectiveness during the sintering of Si_3_N_4_ ceramics. It can provide additional nitrogen atoms to the sintering system, further increasing the N/O ratio of the formed liquid phase. Simultaneously, MgSiN_2_ decomposes at high temperatures, and the released Mg can remove a portion of oxygen atoms in the form of MgO vapor, thereby further purifying the Si_3_N_4_ lattice and reducing the content of intergranular amorphous phase [28,34]. For instance, Fu et al. [29] used Gd_2_O_3_ + MgSiN_2_ as sintering aids and fabricated Si_3_N_4_ ceramics with a thermal conductivity of 124 W·m^−1^·K^−1^ after holding at 1900 °C for 12 h. Similarly, Fan et al. [28] employed YN + MgSiN_2_ as sintering aids, reduced the content of the intergranular phase Y_2_Si_3_O_3_N_4_, and successfully prepared Si_3_N_4_ ceramics with a thermal conductivity of 112 W·m^−1^·K^−1^.

In the sintering process of ceramic materials, the method of introducing sintering aids significantly influences the defects generated in the samples after sintered and the reliability of the final product. The mechanical mixing method, which is currently widely used for raw material blending, only achieves random dispersion between particles. From a microscopic perspective, it is difficult to ensure that each raw material particle effectively contacts the aid particles [30,35]. Furthermore, the use of non-oxide sintering aids leads to a sharp increase in the viscosity of the resulting liquid phase, thereby reducing the efficiency of mass transport. The uneven distribution of sintering aids may cause localized segregation of the liquid phase, which in severe cases can adversely affect the performance of the ceramic. To enhance the uniformity of sintering aid distribution within the starting materials, several coating techniques have been developed, including the formation of coating layers on raw material particles via the decomposition of organic precursors [36], the synthesis of shell structures based on the sol–gel method [37], and the in situ formation of coating layers on particle surfaces using the molten salt method [38,39,40]. Among these, the molten salt method is a versatile synthetic approach that employs molten salts as the reaction medium and offers advantages such as low reaction temperature, short reaction time, controllable process, high degree of reaction, no by-products, and minimal agglomeration of the products [41,42,43]. Compared to solid-state reactions, the diffusion rate of reactants in the molten salt liquid phase is significantly higher than that in the solid state. For example, Shao et al. [38] used metallic Y and SiC as starting materials, with a mixture of NaCl and KCl salts as the molten salt medium, and successfully synthesized Y_3_Si_2_C_2_-uniformly-coated SiC powder after reacting at 1100 °C under an argon atmosphere for 2 h. Similarly, Wan et al. [39] employed metallic Dy and SiC as the starting materials, with NaCl salt as the molten salt medium, and obtained Dy_3_Si_2_C_2_-uniformly-coated SiC powder after reacting at 1100 °C for 1 h. Both types of coated powders exhibited excellent sintering performance, demonstrating that the molten salt method enables the in situ coating of sintering aids and achieves uniform mixing of aids and the starting materials at the microscopic scale.

Given the similarity in sintering mechanisms between Si_3_N_4_ and SiC ceramics, the aforementioned approach can be adopted to synthesize a raw material system comprising α-Si_3_N_4_ coated with sintering aids. It should be noted that a prerequisite for employing this method is that the selected metallic element must be capable of reacting with α-Si_3_N_4_ to form the corresponding sintering aid phase. Currently, Mg is notably the primary metal reported to fulfill this condition. It can react with α-Si_3_N_4_ to form MgSiN_2_ via the reaction shown in Reaction 1. This reaction pathway not only meets the requirement for a low-oxygen-content liquid phase essential for high-thermal-conductivity Si_3_N_4_ ceramics but also achieves uniform coating of the sintering aid on the surface of α-Si_3_N_4_ particles, thereby providing an ideal raw material system for the fabrication of high-performance Si_3_N_4_ ceramics.

3Mg + N_2_ + Si_3_N_4_ = 3MgSiN_2_ (1)

Based on the above analysis, this study aims to successfully construct a MgSiN_2_ coating layer on the surface of α-Si_3_N_4_ particles using the molten salt method with metallic Mg as a precursor, thereby preparing Si_3_N_4_@MgSiN_2_ composite powder with a core–shell structure. The effects of the molten salt system, salt content, and Mg addition amount on the formation of the MgSiN_2_ shell layer were systematically investigated. This study provides a raw material system with low oxygen content and good coating effect, suitable for preparing high-performance Si_3_N_4_ ceramics, and successfully extends the molten salt-assisted coating strategy from the SiC system to the Si_3_N_4_ system.

## 2. Materials and Methods

### 2.1. Preparation of Si_3_N_4_@MgSiN_2_ Powders

The starting materials included α-Si_3_N_4_ (HQ plus, α phase > 93 wt.%, oxygen content < 1 wt.%, size < 10 μm, Alzchem Trostberg GmbH Co. Ltd., Trostberg, Germany), Mg (AR, Kermel Co. Ltd., Tianjin, China), MgSiN_2_ (CX-M02, purify > 99%, Alticera Advanced Materials Co. Ltd., Qingdao, China), NaCl and KCl (AR, Sinopharm Co. Ltd., Shanghai, China). The molten salt used in the experiments was a composite salt consisting of NaCl and KCl in a mass ratio of 1:1. In all samples, the total mass of the salt was fixed at twice the mass of α-Si_3_N_4_. A series of samples were prepared by varying the mass ratio of Mg powder to α-Si_3_N_4_ (specifically 0.200, 0.175, 0.150, 0.125, 0.100, 0.075, 0.067, 0.050, 0.040, and 0.033), and these were labeled sequentially as MS1 through MS10.

The mixtures were homogenized in a planetary ball mill (QM-3S94 Planetary Ball Mill, manufactured by Nanjing Nanda Instrument Factory, Nanjing, China) at 300 rpm for 4 h using silicon nitride balls and a polymer jar, followed by sieving and grinding to obtain uniform starting materials. The as-prepared samples were subsequently placed in MgO crucibles for the subsequent heat treatment process. To investigate the optimal reaction temperature and molten salt system, MS1 samples were heat-treated at 1000, 1100, 1200, 1300, and 1400 °C for 2 h, respectively. Meanwhile, the starting materials with a Mg/Si_3_N_4_ mass ratio of 0.100 were prepared using NaCl, KCl, and NaCl + KCl salt systems and heat-treated at 1300 °C for 2 h. Unless otherwise specified, all other samples were heat-treated at 1300 °C for 2 h. All samples were heat-treated in a pyrolysis furnace under a nitrogen atmosphere at 0.1 MPa, except for the use of nitrogen and argon atmospheres in experiments studying different atmospheric influences. The heat-treated samples were then washed with water, filtered, dried, and sieved to obtain the final products.

### 2.2. Characterizations

Phase composition analysis was conducted using a Cu Kα X-ray diffractometer (XRD, Empyren, PANalytical B.V., Almelo, The Netherlands), with subsequent Rietveld refinement performed via the GSAS-II software. The microscopic morphological features of the products were examined by field-emission scanning electron microscopy (SEM, Hitachi Regulus 8100, Hitachi, Tokyo, Japan) and transmission electron microscopy (TEM, FEI Talos F200X, Thermo Fisher Scientific, Waltham, MA, USA), as well as by energy-dispersive spectroscopy (EDS, Oxford Instruments plc, Abingdon, UK) coupled with TEM. The thermal and mass changes of the samples at high temperatures were analyzed using a simultaneous thermal analyzer (TG-DSC, Netzsch Sta 449 F5/F3 Jupiter, Netzsch-Gerätebau GmbH, Selb, Germany). During the experiment, a nitrogen atmosphere was employed with a gas flow rate of 50 mL·min^−1^. The electronic structure of the surface species on the core–shell structured powder was investigated by X-ray photoelectron spectroscopy (XPS, Thermo Escalab 250Xi, Thermo Fisher Scientific, Waltham, MA, USA). The sintering behavior of the synthesized powder was investigated by observing the sintering deformation of the green body using a visual high-temperature thermal analysis apparatus (TA-Z16B, CTJZH, Tianjin Zhonghuan Technology Co., Ltd., Tijian, China). The shrinkage behavior was measured using in situ shrinkage curves in HPS furnace (ZT-100-18Y, Shanghai Chenhua Science Technology Co., Ltd., Shanghai, China) with a minimal mechanical pressure (3 MPa) applied. All samples subjected to testing were taken from the central region of the product after heat treatment, in order to minimize potential interference from the MgO crucible and atmospheric components on the analytical results.

## 3. Results and Discussion

### 3.1. Synthesis Mechanism of Si_3_N_4_@MgSiN_2_ Powder

**Figure 1:**
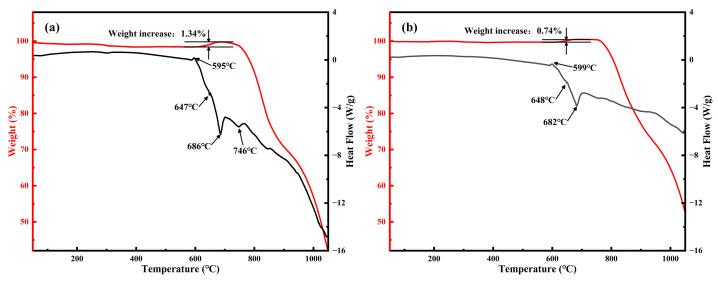
TG-DSC test results of (**a**) MS1 and (**b**) MS5 samples under flowing nitrogen atmosphere.

The TG-DSC analysis results of the MS1 and MS5 samples under a nitrogen atmosphere are presented in Figure 1. The slight weight loss observed below 600 °C is primarily attributed to the removal of adsorbed water and impurities from the system. The weight gain step commencing at approximately 600 °C can be attributed to the nitridation reaction between Mg and atmospheric N_2_, forming Mg_3_N_2_ (Reaction 2) [44], where the incorporation of nitrogen into the system results in an overall mass increase. The exothermic peak appearing at approximately 600 °C in the DSC curve further corroborates the occurrence of Mg nitridation. Within the temperature range of 600–700 °C, the nitridation reaction of Mg proceeds continuously. However, due to the relatively low total Mg content in the system, the heat released from the nitridation reaction is minimal, and consequently, no distinct exothermic peak is discernible in the DSC curve. As the temperature rises to approximately 650 °C, eutectic melting of the NaCl-KCl mixed salt occurs [45], coinciding with a temperature approaching the melting point of Mg, at which point the endothermic peak in the DSC curve sharpens markedly. Simultaneously, the formation of molten Mg and the mixed molten salt enables Mg droplets to disperse and dissolve more thoroughly within the molten salt, substantially increasing the contact area between Mg and the nitrogen atmosphere and thereby accelerating the formation of Mg_3_N_2_. The accelerated weight gain rate observed in the TG curve also indicates an enhanced nitridation reaction rate of Mg, suggesting that the formation of the molten salt liquid phase promotes the nitridation reaction. The endothermic processes within the 650–680 °C interval are primarily associated with the dispersion of the salt and Mg, after which the endothermic trend moderates. By approximately 680 °C, the nitridation reaction of Mg is essentially complete, as evidenced by an inflection point in the DSC curve at this temperature, marking the conclusion of the exothermic reaction. The weight loss and endothermic phenomenon observed after 700 °C are primarily attributable to the gradual volatilization of NaCl and KCl following melting during the TG-DSC measurement. The endothermic peak appearing at around 750 °C can be attributed to the significant acceleration of salt volatilization, which can also be seen in the TG curve. Given that the measurement was conducted under a flowing atmosphere, the volatilized salt was continuously carried away by the flowing gas, preventing the establishment of saturation and leading to persistent salt depletion from the system, thereby resulting in mass loss. Due to the small sample mass employed in the measurement (approximately 10 mg), this volatilization-induced weight loss becomes particularly pronounced. It should be noted that in other experimental processes involving larger sample masses and a static atmosphere, such significant salt loss does not occur.

3Mg + N_2_ = Mg_3_N_2_ (2)

Mg_3_N_2_ + Si_3_N_4_ = 3MgSiN_2_ (3)

**Figure 2:**
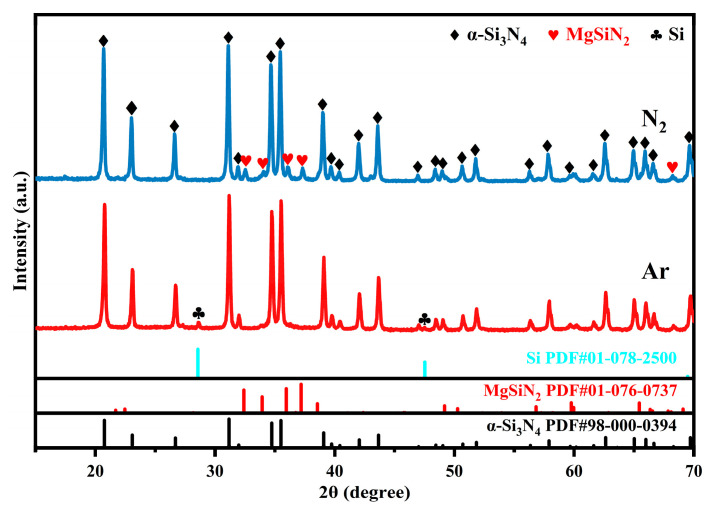
XRD patterns of the MS5 samples after being treated at 1300 °C under different atmospheres for 2 h.

Figure 2 presents the XRD patterns of the MS5 sample after being treated under nitrogen and argon atmospheres, respectively. The analysis reveals that the diffraction peaks intensity of the MgSiN_2_ in the sample reacted in the Ar atmosphere is significantly lower than that the sample under the N_2_ atmosphere, unequivocally demonstrating that N_2_ is an indispensable source of nitrogen for the formation reaction of MgSiN_2_. The XRD pattern of the sample reacted in the Ar atmosphere exhibits distinct diffraction peaks corresponding to Si, which is likely a by-product of the reaction where Mg directly reduces Si_3_N_4_ to form MgSiN_2_. The lower yield of MgSiN_2_ in the Ar atmosphere can be attributed to the high activation energy required for Mg to react directly with Si_3_N_4_, which results in a low reaction extent. Additionally, the absence of N_2_ atmosphere prevents the formation of the intermediate product Mg_3_N_2_ through the nitridation at relatively lower temperatures. This intermediate phase plays a crucial role in immobilizing the Mg element, thereby suppressing its volatilization at elevated temperatures, while simultaneously enabling the reaction with Si_3_N_4_ to form MgSiN_2_ at high temperatures. Consequently, in the Ar atmosphere, Mg rapidly volatilizes and escapes from the reaction system at high temperatures. In conclusion, N_2_ is an essential reactant for the formation of MgSiN_2_.

3MgSiN_2_ = 3Mg + N_2_ + Si_3_N_4_ (4)

**Figure 3:**
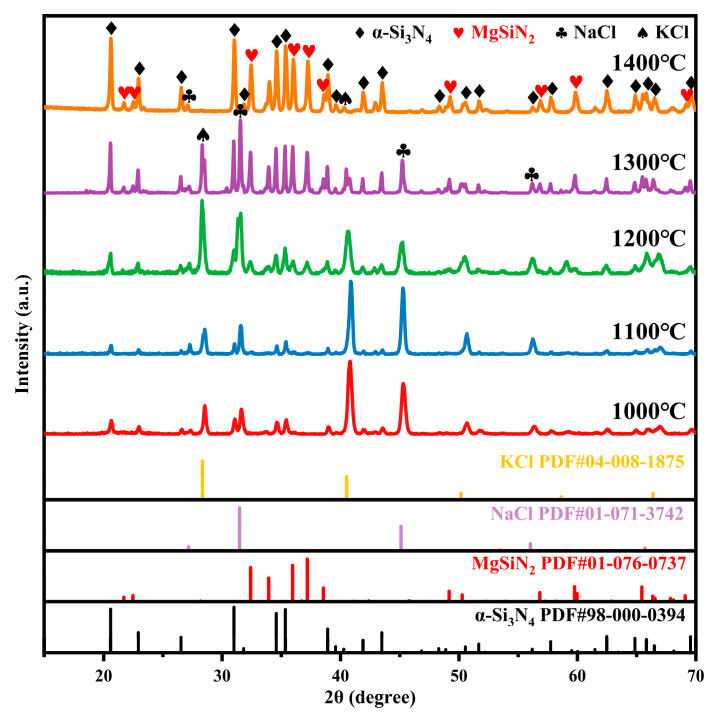
XRD patterns of the MS1 samples after being treated at different temperatures under a nitrogen atmosphere for 2 h without water washing.

The XRD patterns of the products obtained from the MS1 sample after being treated at 1000, 1100, 1200, 1300, and 1400 °C for 2 h (without water washing) are shown in Figure 3. At reaction temperatures of 1000 °C and 1100 °C, the absence of distinct MgSiN_2_ diffraction peaks in the samples indicates that its formation reaction does not proceed under these conditions. As the temperature reached 1200 °C, weak diffraction peaks of MgSiN_2_ emerge, signifying the initiation of MgSiN_2_ formation. However, the reaction rate remained relatively slow due to the low temperature. When the temperature further increases to 1300 °C and 1400 °C, although the relative diffraction peak intensities of MgSiN_2_ and α-Si_3_N_4_ change, the degree of change is minimal compared to previous temperature variations, indicating that the reaction has nearly completed under these temperature conditions. Diffraction peaks attributable to NaCl, KCl, and their resultant solid solutions were clearly detectable in the systems after reacting at 1000, 1100, 1200, and 1300 °C. Nevertheless, the intensities of these salt phase diffraction peaks diminished rapidly with increasing reaction temperature. Notably, after reacting at 1400 °C, diffraction peaks of NaCl and KCl were no longer detectable in the XRD pattern. This phenomenon is primarily attributed to the volatilization behavior of the molten salt. According to the Clausius-Clapeyron equation, the vapor pressure of the eutectic melt formed by the NaCl-KCl salt mixture at approximately 650 °C increases exponentially with rising temperature, leading to a drastic acceleration in its volatilization rate. Consequently, as the holding temperature increases, the volatilization rate of the salt accelerates, resulting in a gradual decrease in the intensity of its diffraction peaks. Given that the boiling points of NaCl and KCl (~1413 °C and ~1420 °C, respectively) are very close to the holding temperature of 1400 °C, prolonged exposure at this temperature is sufficient to volatilize nearly all of the salt, leaving essentially no salt residue in the final product.

**Figure 4:**
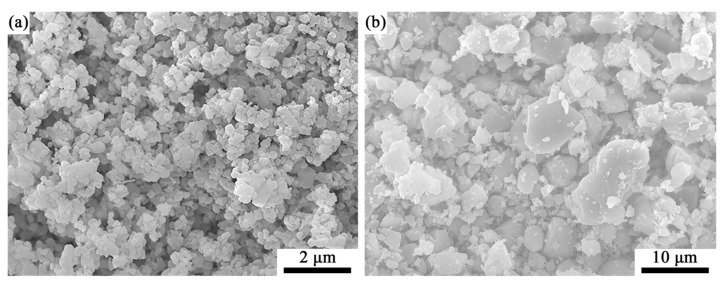
SEM images of pure phase MgSiN_2_ samples after being treated at (**a**) 1300 °C and (**b**) 1400 °C for 10 min.

The SEM micrographs of the pure phase MgSiN_2_ samples after being compressed into discs under a pressure of 10 MPa and treated at 1300 °C and 1400 °C for 10 min are shown in Figure 4a,b, respectively. The sample treated at 1300 °C retains a particulate morphology at the submicron scale. In contrast, after treatment at 1400 °C the sample exhibits significantly coarsened, larger particles. This morphological difference can be attributed to the significantly enhanced surface diffusion activity of MgSiN_2_ in the temperature range of 1300–1400 °C. When the temperature rises to 1400 °C, particles may undergo coarsening through surface diffusion and Ostwald ripening mechanism. At the same time, under conditions close to their thermal decomposition temperature [46], local decomposition redeposition processes may also promote grain growth. Based on these observations, to achieve a uniform and fine MgSiN_2_ coating layer on the Si_3_N_4_ particles, the heat-treatment temperature should not exceed 1300 °C. Furthermore, it has been reported that MgSiN_2_ undergoes a decomposition reaction above 1400 °C (Reaction (4)). Therefore, considering account morphological control, coating uniformity, and thermal stability, 1300 °C is selected as the optimal temperature for the preparation of the MgSiN_2_ coating layer.

**Figure 5:**
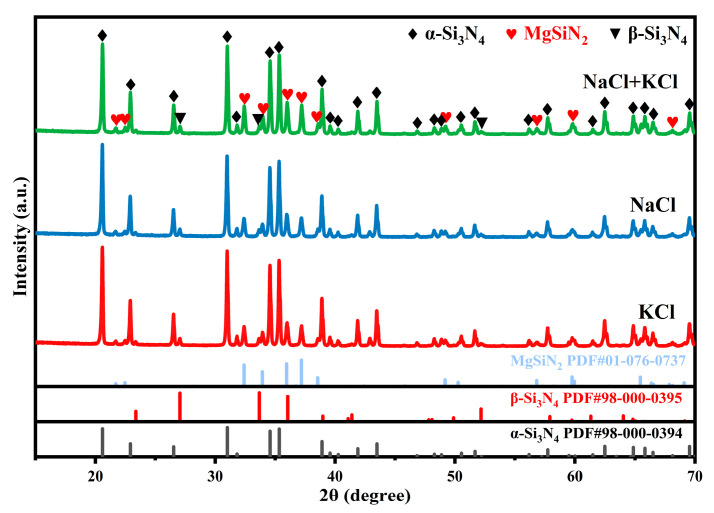
XRD patterns of the samples with Mg/Si_3_N_4_ mass ratio of 0.100 after being treated under different molten salt environments.

Figure 5 presents the X-ray diffraction patterns of the samples with a Mg/Si_3_N_4_ mass ratio of 0.100 after being treated in different molten salt environments. The phase fractions obtained through structural refinement of the XRD results are summarized in Table 1. The analysis indicates that, compared to samples processed using NaCl or KCl alone as the molten salt medium, the sample treated in the NaCl-KCl mixed molten salt exhibits significantly enhanced formation of MgSiN_2_. This phenomenon can be attributed to the lower eutectic melting point of the mixed salt system, which enables the establishment of a stable molten salt liquid phase environment at a relatively low temperature. Such an environment facilitates the uniform dispersion of Mg and its effective contact with N_2_, thereby accelerating the nitridation reaction of Mg, reducing the volatilization loss of Mg at elevated temperatures, and ultimately promoting the formation of MgSiN_2_.

### 3.2. Controlled Synthesis and Microstructural Analysis of MgSiN_2_ Shells

**Figure 6:**
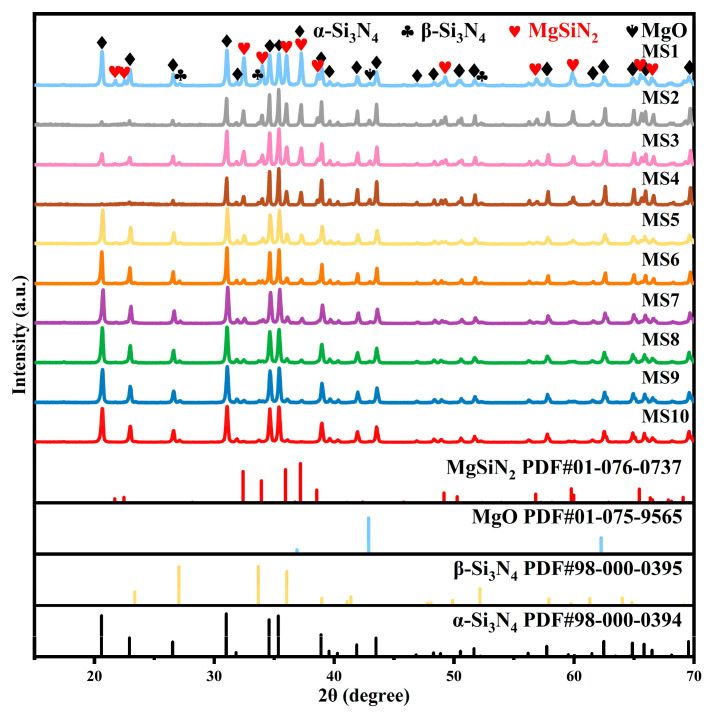
XRD patterns of the samples with different Mg/Si_3_N_4_ mass ratios after being treated at 1300 °C for 2 h.

Figure 6 shows the phase composition of the samples obtained from the starting materials with different Mg/Si_3_N_4_ mass ratios after being treated at 1300 °C for 2 h followed by water washing. The results indicate that diffraction peaks corresponding to the MgSiN_2_ were clearly detected in all the samples investigated. As the Mg content in the starting materials decreased, the diffraction peak intensity of MgSiN_2_ in the products gradually weakens. This demonstrates that the yield of MgSiN_2_ in the final products can be effectively controlled by precisely adjusting the Mg/Si_3_N_4_ mass ratio in the starting materials.

**Figure 7:**
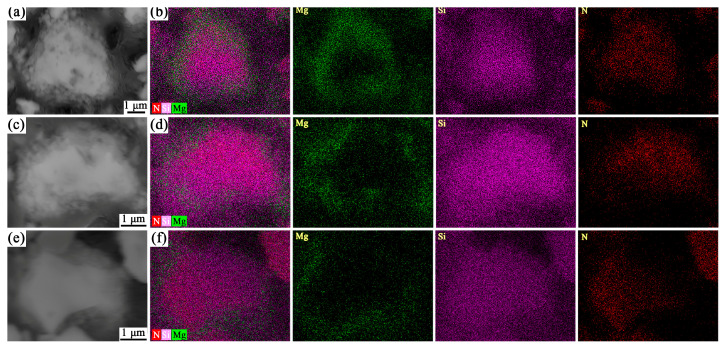
Cross-sectional SEM images and corresponding elemental mapping of the samples with different Mg/Si_3_N_4_ mass ratios: (**a**,**b**) MS4, (**c**,**d**) MS5, (**e**,**f**) MS6.

The cross-sectional microstructures of the MS4, MS5, and MS6 samples treated at 1300 °C for 2 h were characterized, with the results presented in Figure 7. It can be observed that the surfaces of the Si_3_N_4_ particles in all samples were covered with a substantial amount of finely dispersed particles. As the Mg/Si_3_N_4_ mass ratio in the starting materials increased, the quantity of these surface-attached particles increased significantly, which consequently led to an increase in the surface roughness of the Si_3_N_4_ particles. The corresponding elemental mapping results indicate that the interior region of the particles consisted of Si and N elements, aligning with the composition of α-Si_3_N_4_, while the periphery region of the particles was coated with a layer primarily composed of Mg, Si, and N elements. Furthermore, with increasing Mg content in the starting materials, the thickness of this coating layer exhibited a progressively increasing trend. This is extremely similar to the SEM image of the sample cross-section shown in reference [38,47], which also demonstrates the feasibility of this method in the Si_3_N_4_ system. Integrating the results from XRD phase analysis and SEM-EDS elemental mapping, it can be concluded that the reaction in the molten salt environment leads to the formation of a new MgSiN_2_ phase on the surface of the Si_3_N_4_ particles, successfully constructing a core–shell structure with Si_3_N_4_ as the core and MgSiN_2_ as the shell.

**Figure 8:**
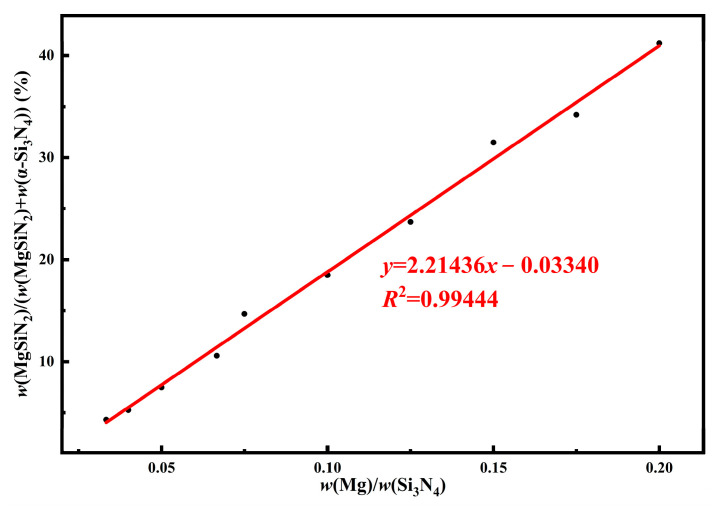
Regression analysis between the Mg/Si_3_N_4_ mass ratio and the mass fraction of MgSiN_2_ (calculated as w(MgSiN_2_)/(w(MgSiN_2_) + w(Si_3_N_4_))), with the corresponding regression equation.

To quantitatively analyze the effect of the Mg/Si_3_N_4_ mass ratio in the starting materials on the formation of MgSiN_2_ in the products, the obtained XRD patterns were refined to determine the mass fractions of each crystalline phase in the products, and the results are summarized in Table 2. The data reveal that as the Mg/Si_3_N_4_ mass ratio in the starting materials decreases, the mass fraction of Si_3_N_4_ in the products gradually increases, while the mass fraction of MgSiN_2_ correspondingly decreases. A regression analysis was conducted between Mg/Si_3_N_4_ mass ratio and the mass fraction of MgSiN_2_ in the product (calculated as the proportion of MgSiN_2_ relative to the combined weight of α-Si_3_N_4_ and MgSiN_2_). The results are presented in Figure 8. The regression analysis indicates a highly significant linear positive correlation between the Mg/Si_3_N_4_ mass ratio in the starting materials and the mass fraction of MgSiN_2_ in the product. Consequently, precise adjustment of the relative content of Mg in the starting materials enables accurate control over the yield of MgSiN_2_ in the final product. This finding further implies that the MgSiN_2_ coating layer on the Si_3_N_4_ particle surfaces in the final composite powders can be tailored by adjusting the starting materials composition, thereby meeting the design requirements for different amounts of sintering aids or specific interface characteristics.

Furthermore, the integrated results from Figure 6 and Table 2 indicate that a small amount of β-Si_3_N_4_ phase is present in all the resultant products. Its sources include a small amount of β-Si_3_N_4_ present in the initial α-Si_3_N_4_ raw materials, as well as a small amount of α-Si_3_N_4_ that has undergone the α → β phase transformation at high temperatures. Concurrently, a certain amount of the MgO phase is also observed in all samples. This may originate from the trace MgO present in the Mg powder. Moreover, due to the intrinsic structural defects in α-Si_3_N_4_, a certain amount of oxide layer in the form of SiO_2_ is present on its surface [48]. Such oxygen species exhibit high reactivity and tend to react with Mg at elevated temperatures, forming MgO that remains in the system, which constitutes another significant source of MgO.

### 3.3. Surface Structure of Si_3_N_4_@MgSiN_2_ Particles

**Figure 9:**
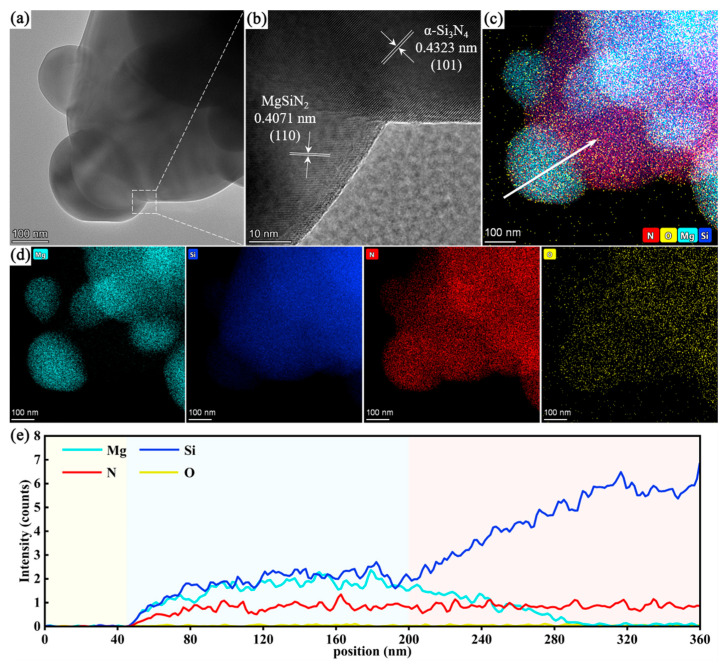
(**a**,**b**) TEM images of the MS5 sample, (**c**,**d**) corresponding elemental mapping of the TEM images, (**e**) EDS line-scan profile along the white arrow in (**c**).

To further investigate the formation and attachment morphology of MgSiN_2_ on the surface of Si_3_N_4_ particles, high-resolution transmission electron microscopy (HRTEM) analysis was performed on the as-synthesized MS5 samples, with the results presented in Figure 9. Two distinct sets of lattice fringes are clearly identifiable in the HRTEM image. The measured interplanar spacings are 0.4323 nm and 0.4071 nm, corresponding to the (101) plane of α-Si_3_N_4_ and the (110) plane of MgSiN_2_, respectively. Figure 9c,d show the corresponding TEM-EDS elemental mapping results. The distributions of Mg, Si, and N elements further confirm the spatial relationship between the MgSiN_2_ and Si_3_N_4_ phases. In Figure 9e, the EDS line-scan profile collected along the white arrow indicated in Figure 9c reveals the variation in elemental content. The intensity of the Mg signal gradually decreases from the exterior toward the interior of the particle, indicating that the reaction between Mg and Si_3_N_4_ within the molten salt environment proceeds progressively from the surface inward. This reaction pathway aligns well with the classical “template growth” mechanism associated with molten salt synthesis [38,47,49].

**Figure 10:**
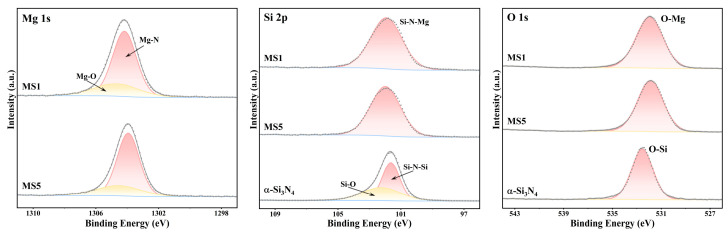
XPS spectra of the Mg 1s, Si 2p, and O 1s for the MS1, MS5, and α-Si_3_N_4_ samples.

To further investigate the chemical states on the surface of the Si_3_N_4_@MgSiN_2_ particles and the effect of Mg on oxygen impurities in the starting materials, X-ray photoelectron spectroscopy (XPS) analysis was performed on the MS1, MS5, and raw α-Si_3_N_4_ samples, with the results presented in Figure 10. Analysis of the Mg 1s spectra indicates that on the surface of the Si_3_N_4_@MgSiN_2_ particles, Mg exists predominantly in the form of Mg-N bonds, which aligns with the structural characteristic of Mg occupying the tetrahedral MgN_4_ coordination center in MgSiN_2_ [50]. In the Mg 1s spectra of the MS1 and MS5 samples, a minor amount of Mg atoms was detected in the form of Mg-O bonds. Correspondingly, peaks corresponding to O-Mg bonds are observable in their O 1s spectra, and no peaks corresponding to Si-O bonds were detected in the Si 2p spectra of these two samples. In sharp contrast, a relatively distinct peaks corresponding to Si-O bonds were present in the Si 2p spectrum of the raw α-Si_3_N_4_ sample, and its O 1s peaks corresponded to the binding energy of O-Si bonds. This difference confirms the gettering effect of Mg on oxygen impurities in the raw α-Si3N4. In the Si 2p spectra, the characteristic peaks corresponding to Si-N bonds for the MS1 and MS5 samples is located at 102.0 eV, which is assigned to Si-N bonds within the MgSiN_2_ phase. This represents a positive shift of 0.3 eV compared to the peaks corresponding to standard Si-N bonds in α-Si_3_N_4_ at 101.7 eV. This shift is primarily attributed to the strong electron-withdrawing inductive effect exerted of Mg on N in MgSiN_2_. This effect reduces the shielding of the electron cloud around adjacent Si atoms by N atoms, leading to an increase in the binding energy of the Si 2p electrons and consequently a stronger Si-N bond in MgSiN_2_. This phenomenon provides evidence at the electronic structure level that the reaction between Si_3_N_4_ and Mg on the surface of the Si_3_N_4_ particles leads to the formation of MgSiN_2_.

### 3.4. Sintering Properties of Si_3_N_4_@MgSiN_2_

**Figure 11:**
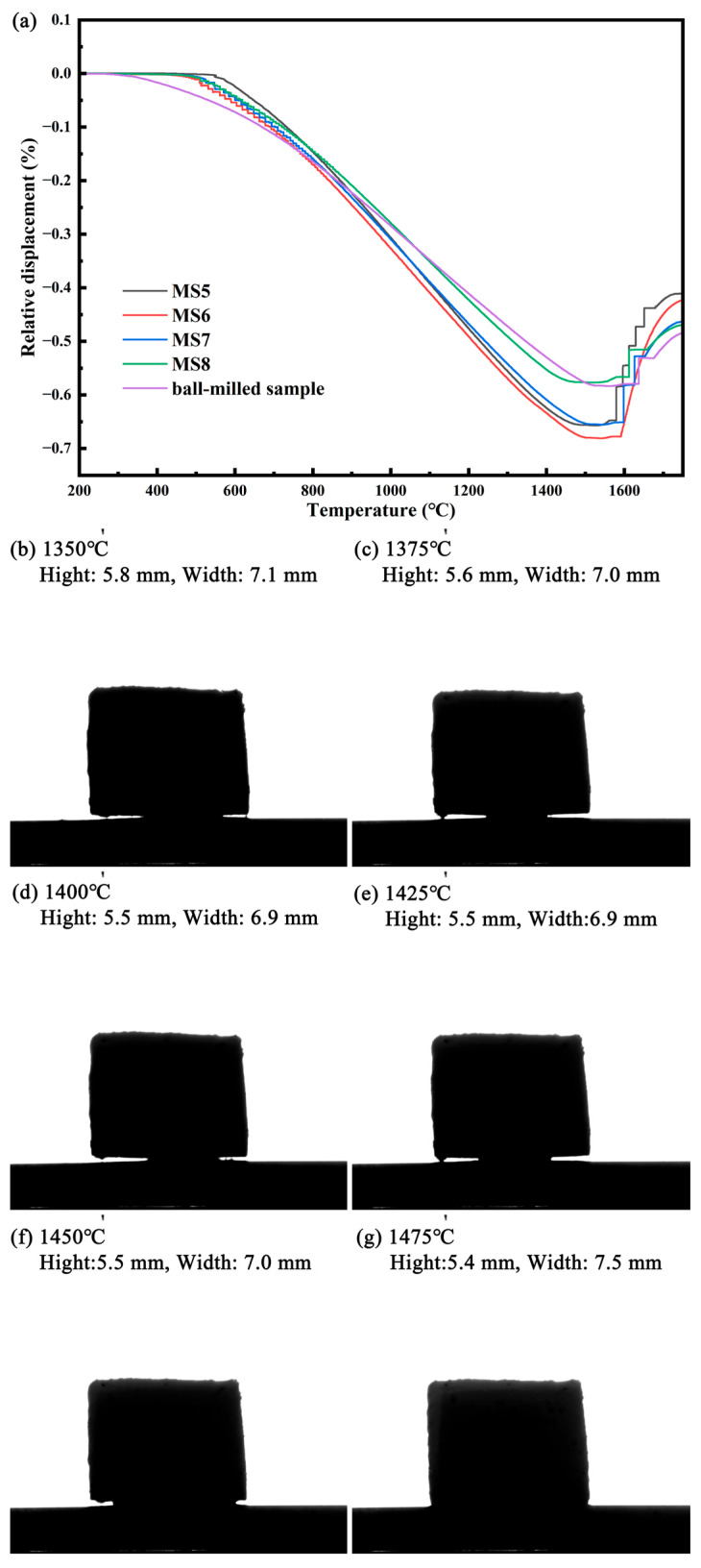
(**a**) Sintering shrinkage curves of the five samples during the sintering process, (**b**–**g**) images of the MS5 sample at different temperatures during the in-situ visualization of the sintering process.

The shrinkage behavior of samples MS5–MS8 and a Si_3_N_4_ raw material mixture prepared by ball milling with the addition of 7 wt.% MgSiN_2_ as a sintering aid was investigated during the sintering process, with the results shown in Figure 11a. It can be observed that the shrinkage cessation temperatures of the four MS5–MS8 samples are lower than that of the ball-milled sample, indicating a lower temperature for the onset of liquid phase formation. Simultaneously, visual sintering observations of the MS5 sample were conducted under a nitrogen atmosphere, as presented in Figure 11b–g. Figure 11b reveals that when the temperature reaches approximately 1350 °C, the sample height exhibits a decreasing trend with further temperature increase, suggesting the onset of melting around this temperature. Based on the images of the sample at various temperatures shown in Figure 11c–g, distinct wetting behavior of the sample toward the substrate can be observed at approximately 1425 °C. These phenomena demonstrate that the Si_3_N_4_@MgSiN_2_ powder can melt to form a liquid phase at a relatively low temperature and maintain this liquid state over a wide temperature range. This characteristic is highly favorable for promoting the α → β phase transformation, as well as facilitating the nucleation and grain growth of β-Si_3_N_4_ during the sintering process of Si_3_N_4_ ceramics [13,14,51].

## 4. Conclusions

In summary, Si_3_N_4_@MgSiN_2_ composite powder with a core–shell structure was successfully synthesized via the in situ reaction between Mg and α-Si_3_N_4_ in a molten salt environment. The reaction pathway involved nitridation of Mg at approximately 600 °C to form Mg_3_N_2_, which dispersed in NaCl-KCl molten salt and reacted with α-Si_3_N_4_ above 1200 °C via a template growth mechanism, forming a well-crystallized MgSiN_2_ shell. Temperatures of 1200 °C or below were insufficient for MgSiN_2_ formation, while 1400 °C led to grain coarsening. In contract, the MgSiN_2_ layer formed at 1300 °C exhibits the optimal morphology and uniformity, thereby establishing this as the optimal reaction temperature. Furthermore, investigation into different molten salt systems reveals that, compared to using a single salt, the use of mixed salts can more effectively facilitate mass transfer and contact between reactants, which is more conducive to the formation of MgSiN_2_. A linear positive correlation was established between the coating thickness and the Mg/Si_3_N_4_ mass ratio in the starting materials. This relationship enables the controllable preparation of core–shell powders with varying shell thicknesses through precise adjustment of the starting material composition. The synthesized powder exhibited liquid phase formation at approximately 1350 °C, thereby conferring higher sintering activity to the entire system. This study provides a comprehensive synthesis strategy and mechanistic understanding for the controllable preparation of Si_3_N_4_@MgSiN_2_ core–shell structured materials, offering a new material foundation for the synthesis of Si_3_N_4_ ceramics with high thermal conductivity.

## Figures and Tables

**Table 1:** The content of each phase in samples prepared using different salt systems.

Sample	Types of Salt	Content of Each Phase/wt.%		
		α-Si_3_N_4_	β-Si_3_N_4_	MgSiN_2_
**1**	**KCl**	**77.922**	**6.094**	**15.894**
**2**	**NaCl**	**76.076**	**6.206**	**17.718**
**3**	**NaCl + KCl**	**72.228**	**5.794**	**21.978**

**Table 2:** The phase content of samples with different Mg/Si_3_N_4_ mass ratios after sintering.

Sample	Mg/Si_3_N_4_ Mass Ratio	Content of Each Phase/wt.%				w(MgSiN_2_)/(w(MgSiN_2_) + w(α-Si_3_N_4_))
		α-Si_3_N_4_	β-Si_3_N_4_	MgSiN_2_	MgO	
MS1	0.200	57.207	1.849	40.103	0.841	41.212
MS2	0.175	62.258	2.963	32.332	2.447	34.181
MS3	0.150	65.034	3.465	29.872	1.629	31.475
MS4	0.125	72.228	2.788	22.417	2.567	23.685
MS5	0.100	78.991	2.334	17.915	0.760	18.487
MS6	0.075	80.732	3.720	13.884	1.664	14.674
MS7	0.067	85.987	2.925	10.185	0.903	10.590
MS8	0.050	89.385	2.610	7.228	0.777	7.481
MS9	0.040	91.801	2.604	5.098	0.497	5.261
MS10	0.033	90.809	3.296	4.096	1.798	4.316

## Data Availability

The original contributions presented in this study are included in the article. Further inquiries can be directed to the corresponding authors.
